# Book Reviews

**Published:** 1806-10

**Authors:** 


### CRITICAL ANALYSIS

OF THE

### RECENT PUBLICATIONS

ON THE

### DIFFERENT BRANCHES OF PHYSIC, SURGERY, AND MEDICAL PHILOSOPHY.

---

*Medical Collections on the Effect of Cold as a Remedy in certain Diseases; with an Appendix, containing an Account of the Experiments made with a View to ascertain the Effect of cold Water on the Pulse.* By JOHN EDMONDS STOCK, M. D. Licenciate of the Royal College of Physicians in London, &c. &c. and Physician in Bristol. 8vo. pp. 200.

THE application of cold, particularly the affusion of cold water, has for some time become so important a remedy, that different writers have put in their claims for the discovery, or rather the revival of so apparently bold a practice. Dr. Stock carries his pretensions no further back than the year 1797, when he printed his Inaugural Dissertation on that subject. He now, with equal modesty, offers principally a compilation of what has been done by others.

Chapter the first is on the general effect of cold on the human body. As it is of the utmost importance to state the leading theory of every author in the most precise terms, we always prefer giving his own words, if not too diffuse for our limits.

Cold is considered by Dr. Stock as a constantly sedative remedy. The following are his " reasons for adopting this opinion. They are derived, First, from the paleness and contraction of the skin, which succeed the application of Cold.—Secondly, from its diminishing or weakening the action of the heart, and arteries.—Thirdly, from the debility and inactivity observable in the inhabitants of cold countries.—Fourthly, from the gradual diminution of the vital powers, which commences with its first application, and which, if its operation be long continued, terminates in their entire extinction, either in particular parts, or in the whole body.—And lastly, from the accumulated excitability which it induces to the stimulus of heat.

" A variety of circumstances may so modify the effects of any given degree of temperature, that it would be impossible to fix the point where actual Cold commences. But, it may be sufficiently accurate for our present purpose, to observe, upon the authority of various writers, that the body constantly retains its natural temperature in a healthy man, when Fahrenheit's thermometer stands at 62 degrees. A temperature inferior to this, unless its operation be counteracted by some mental or coporeal stimulus, gradually abstracts the sensible heat of the body.

" The

"The first general effect of this diminished temperature is a paleness of the skin, produced by the contraction of the superficial vessels. If the degree of Cold be considerable, this effect is particularly evident. When Dr. Solander was exposed to the intense frosts of Terra del Fuego, his feet were so contracted, during the slumber of a few minutes, that his shoes fell off, when he was compelled to rise. This is called by Dr. Cullen, its astringent quality. The epithet is probably misapplied. An extensive system of vessels is reduced to a state of quiescence, and the effect is obvious in proportion to the extent of surface exposed. This exposure causes in a given time, an increased flow of urine, which is supposed by a late eminent Physiologist, to be owing to the diminished action of the cutaneous absorbents, and the inverted motions of those of the bladder. Whatever be the theory of its operation, the fact will admit of important application hereafter.

"A second effect of this diminished temperature, is a weakened action of the heart and arteries, which is more particularly obvious, if the Cold applied, be either violent in its degree, or long in its duration. This fact is proved by various examples. In cold countries the pulse is uniformly slow. In Greenland it seldom beats above forty strokes in a minute. Similar effects are produced by the general or partial application of cold water; a decisive example of which is recorded by Dr. Rush, in his account of the Yellow Fever of Philadelphia, in 1793. "In an experiment," says he, "which was made at my request by one of my pupils, by placing his feet in cold pump water for a few minutes, the pulse was reduced 24 strokes in a minute, and became so weak as hardly to be perceptible."

"Experiments upon the effects of cold water on the pulse, have been also made by Dr. Marcard, first Physician to the Duke of Holstein, with results precisely similar. After it had been applied for about four minutes, he observes that the pulsations were uniformly much diminished, both in force and frequency.

"The debility and inactivity observable in the inhabitants of cold countries, were enumerated in the third place, among the effects of Cold. These effects become evident, in proportion as we advance to the frozen regions which encircle the Pole. All travellers who have penetrated into those inhospitable climes, have given us a corresponding account of the torpid and feebly animated existence of their inhabitants. This debilitating effect has been extended by some writers to the faculties and operations of the mind; and the inhabitants of the Frigid Zone have been accused by them of stupidity, imbecility, and cowardice.

"Fourthly,—Violent degrees of Cold, if long applied, are, as is well known, capable of entirely extinguishing the vital powers. Seven thousand Swedes are said to have perished at once, in attempting to pass the frozen mountains, which form the western barrier between that country and Norway. But Cold, when combined with moisture, chills the body much faster than dry Cold of a much lower temperature; for water is a much better conductor of of heat than air. If combined with the moisture produced by the solution of snow or hail, even though it be but a few degrees lower than the freezing point of Fahrenheit, it may prove fatal. The evaporation which takes place gradually carries off the heat of the body, till there no longer remains a sufficient degree of it, for the support of animal life. In such cases, the sufferer feels himself exceedingly chill and uneasy; he gradually becomes unwilling to use exercise, or to keep himself warm, and at last feels drowsy; sits down to refresh himself with sleep, and awakes no more.

"Fifthly,—The application of Cold to the surface of the body, produces accumulated sensibility to the stimulus of heat. Hence the glow upon the skin after immersion from the cold bath, or upon entering an apartment moderately heated, after exposure to a refrigerated atmosphere. From the accumulated sensibility thus produced, a debilitated frame has experienced renovated energy; and the observation of this effect has, I believe, been a principal source of the belief that cold has a tonic and stimulant operation. But a satisfactory answer to any objections arising from this source, is, I think, furnished by the ingenious explication of these supposed stimulant effects, given by Dr. Brown, and Dr. Darwin. "If Cold," observes the Author of the Elements of Medicine, "sometimes appears to stimulate, it produces that effect not as actual Cold; but, either by diminishing excessive heat, and reducing it to its proper stimulating temperature, or, by accumulating the excitability diminished by excessive stimulus, and communicating energy to the stimulus of the exciting powers, now acting too languidly."

"Dr Darwin's explanation is, perhaps, still more satisfactory. 'From the quiescence,' he observes, 'of such extensive systems of vessels as the glands and capillaries of the skin, and the minute vessels of the lungs, with their various absorbent series of vessels, a great accumulation of sensorial power is occasioned; part of which is again expended in the increased exertion of all these vessels, with an universal glow of heat in consequence of this exertion, and the remainder of it adds vigour to both the vital and voluntary exertions of the whole day.'

"If the activity of the subcutaneous vessels, and of those with which their actions are associated, was too great before cold immersion, as in the hot days of summer, and by that means the sensorial power was previously diminished, we see the cause why the cold bath gives such present strength, namely, by stopping the unnecessary activity of the subcutaneous vessels, and thus preventing the too great exhaustion of sensorial power."

The only objection we can make to this is the concluding part, in which cold is said to prove tonic and stimulant, by producing accumulated sensibility to the stimulus of heat, in the *Author's* words; "accumulated excitability" in *Dr. Brown's* words; and "accumulation of sensorial power" in *Dr. Darwin's* words.— Whenever we meet with these kind of expressions we are always on the alarm, lest we should be led astray; and whilst we are looking looking for one thing, should overlook another. As to the glow which follows the application of cold, it would to us seem more easily explained by admitting that, under exposure to cold, the vessels are stimulated to generate heat, in proportion to the necessity for it; and that the action of generating heat being once excited, continues for a time after the immediate cause is removed. This is the more probable, because if the application of intense cold is continued beyond a certain time, the powers of the vessels are no longer able to resist it by generating heat, and the actions of life cease, never to be restored. It should also be recollected, that this renovated heat is greater in sensation than in reality, because after exposure to a certain degree of cold, the temperature of the atmosphere will feel warmer, though in reality the same. Hence a more than ordinary glow after the surface of the body has ceased to be exposed to a colder temperature.

That the bare "accumulation of sensibility to the stimulus of heat," or "accumulation of excitability," or "preventing the too great exhaustion of the sensorial power," are not the only causes of the "renovated energy experienced by the debilitated frame," we may fairly presume, by reflecting that a bath heated somewhat above the degree of the surrounding atmosphere is attended with all these advantages, and not merely for an instant, but as permanently as any received from the cold bath. Now, if this be admitted, and we believe in Dr. Stock's neighbourhood such an opinion is gaining ground, may it not reconcile that gentleman to Dr. Currie's theory, that cold, though sedative when applied in an extreme degree, may be stimulant in a lighter degree? And this is further proved by the state of the subject to whom it is applied. If a man in high health remains for a certain period in the cold bath, the consequence is a subsequent glow. If he is in reduced health, this glow is less certain; and it is found advisable to exhibit the tepid bath. Under circumstances of fever, if high vascular action is excited, and heat generated beyond the standard of health, the heat will be moderated by the cold water, the action lessened, and the patient feel a relief in proportion to the effect produced, and the previous necessity. Nor does increased action necessarily follow, because the necessary stimulus will extend no further than to excite the standard heat; and if the patient is relieved by abstraction of the former heat and the lessening of the vascular action, there is no reason why this relief may not remain for some time; after which the remedy may be repeated.

We have thought it right to premise these remarks, after which we shall, as seldom as possible, interrupt the Author in his narrative or opinions, leaving the reader to judge between us.

The chapter concludes with some ingenious remarks on the effect of charcoal fumes, in which the effects of carbonic acid gas are shewn to be stimulant, and, in consequence of that property, to produce their effect on the brain; but though nothing else has been been discovered in the fumes of charcoal, yet as their effects on the human body are not exactly similar to those of gas extricated by effervesence or fermentation, so we are not perfectly satisfied that they contain no other properties.

The Second Chapter contains Remarks on the History of the Medical Application of Cold, and its Effects. In this history we seem to follow the subject from the writings of Hippocrates, Galen, Celsus, Sir J. Floyer, Drs. Baynard, Moore, Currie, Cullen, Brown, Darwin, Aikin, Brydone. The practitioners in the Yellow Fever, two of the army physicians, Drs. Senirot and Sutton, are quoted by Dr. Beddoes, as attesting, that during the rapid retreat of the British troops in Flanders, fever patients carried in open waggons all recovered. Amidst this variety of names, we in vain looked for Dr. R. Jackson's. It would not have been necessary to offer this anecdote of the British troops at second hand, and by report, had our author attended to that truly valuable writer. He might also have dated the application of cold, somewhat earlier than Mr. Moore, and even of cold water, before that gentleman proposed cold air. Whether this omission is designed, or accidental, we pretend not to determine it. In either case, we regret that Dr. Stock has not availed himself of the original ideas which are offered by Dr. Jackson, nor at least given him a just claim to his share in restoring and confirming this practice.

Some useful cautions on the application of so powerful a remedy are interspersed, and the Author concludes this chapter by informing his readers, that in tracing the beneficial effects of Cold in various diseases, he shall pursue as nearly as possible, "the nosological arrangements of Dr. Cullen; notwithstanding those defects peculiar to itself, from the prejudices of its celebrated author." This is following one's master with a vengeance. It may be very proper, for any thing we know, but in a subject, every part of which requires so much accuracy as the history and treatment of diseases, we know not how an author can proceed with an imperfect system of nosology. We know not, indeed, why he should incumber himself with any system, as all are said to be imperfect; but still less, why he should choose one which has imperfections peculiar to itself.

The Third Chapter is on the use of cold applications in several genera of febres of Dr. Cullen. First, in intermittent fever. This contains some remarks on the phænomena of perspiration, on Dr. Darwin's theory, and some cases in which cold was applied in tertians. As the management of tertians in general is pretty well understood in this country, and as these cases will not particularly illustrate those anomala which sometimes occur, we shall pass over this part of the subject. We are next shown its use in synocha, our remarks on which we shall reserve to the conclusion of our Analysis. On typhus the author is more minute, but, as might be expected, finds himself dreadfully hampered on the subject of yellow fever, which is considered by some of the Nosologists gists as a variety of typhus. How much we regret that Dr. Stock has not given, in this place, his own definition of typhus.* Dr. Currie's cautions, relative to the temperature, are very properly enforced; the use of cold in yellow fever is considered, and a candid inquiry is instituted, how far the application of this remedy is forbidden by the application of mercury.

The Fourth Chapter is on the use of cold in the phlegmasiæ. Here the author passes through inflammation of most of the viscera, and shows, by authority or analogy, the advantage of cold applications in many of them. The chapter concludes with some judicious remarks on the Controversy concerning cold water in gout.

Chapter V. Of the use of cold in the exanthemata. In the first of these (the small-pox) the practice has been so long established as to require no further arguments than such as would authorize cold ablutions. On the measles, the question has been more disputed, but is here candidly discussed. The author next shows, with much judgment, that critical eruptions are not checked by cold; and that advantage is derived from its free use in scarlatina. In these the author, with much propriety, proposes sponging rather than the shock of cold affusion. The plague, erysipelas, prickly heat, follow in order, and the chapter concludes with some excellent observations on the erroneous terrors sometimes apprehended on *drying up* cutaneous eruptions

Chapter VI. Of the use of cold in hæmorrhage. Chap vii. In profluvia. This is confined principally, in the present chapter, to dysentery.

Chapter VIII. On the use of cold in comata. In this chapter the author finds a difficulty in reconciling the Brunonian language of indirect debility, when applied to the effects of drunkenness. We confess we find many more difficulties, but the one proposed by Dr. Stock is enough, when we reflect how peculiarly the phenomena are calculated to illustrate the whole of that *simple theory.*

Chapter IX. Of the use of cold in spasmi, contains, principally, authorities for or against its use in tetanus; convulsio and asthma; epilepsy and colic; ileus and cholera; hysteria; hydrophobia.

Chapter X. On the use of cold in vesaniæ. Chap. xi. On the use of cold in tympanites and dropsy. Chap. xii. gives some good cases of ischuria. Chap xiii. On the use of cold in burns.

Chapter XIV. Contains a summary review of the whole. This affords many useful remarks; but we in vain looked for one important consideration, which occurred to us in almost every page from the time we promised our remarks on Synocha, but to which the author has not thought it necessary to give that attention, which appeared to us absolutely necessary. We shall briefly state our

* In another part of the work, some good observations are inserted on the misapplication of that word.

our own opinion, and leave the reader to determine for himself. The whole class of inflammatory diseases, are for the most part attended, not only with increased vascular action, but with plethora. Even where the latter does not generally exist, it must partially; and our business, where an important organ is pursuing a wrong action, is, to check that action as early as possible, before permanent mischief may ensue. In common cases, these actions continue for a time, and cease spontaneously. In these it is of little consequence, whether we sponge, immerge, immerse, or omit them all. In more serious cases, when by a concurrence of symptoms, we are convinced that the disease, if not soon removed, must end in suppuration or gangrene; we must not confine ourselves to cold water, but bleed with a freedom proportioned to the exigency of the case. Cold water in external inflammation, is a valuable assistant. In internal cases, we are not prepared to speak of its merits.

The Appendix contains, 1st. A Case of obstinate Head-Ach, cured by drinking six quarts of cold water daily, for two or three months. The author suspects that this habitual head-ach might have been the effect of habitual potations. 2d. The result of experiments, made with a view to ascertain the effects of cold water on the pulse. 3d. A note on the gout, relating to a subject, of which some of our readers have read enough, and those who have not, will wish to consult the whole. 4th. A case of epilepsy, which was uncured when the work was printed. On the whole, we consider this work as a useful compilation; the modesty of the author, in keeping himself so entirely in the back ground, precludes any severity of criticism, and induces us to encourage him, on any future occasion, to speak more from himself, and less from others.

---

*A Treatise on Vaccine Inoculation;* by Robert Willan, M. D. F. A. S. Physician Extraordinary to the Fever Institution, and to the Public Dispensary in London.

It is with much pleasure we find Dr. Willan calling the public attention to this important subject, at a time when it should, in a particular manner, receive every assistance before it is submitted to the august and impartial decision of a national tribunal.

The first section of his work contains a recapitulation of the experiments hitherto made on the combined inoculation of the variolous and vaccine fluids. The controversy concerning vaccine pustules or vesicles, and on the possibility of an hybrid disease, is ably and clearly discussed.

The second section is "on the Characteristics and Effects of perfect Vaccination." This is so important a part of the subject, that we cannot withhold transcribing it.

"Vaccination is accounted perfect, when recent lymph has been carefully inserted beneath the cuticle, in a person free from any contagious disorder, and has produced a semi-transparent, pearl-coloured vesicle, which, after the ninth day, is surrounded by a red areola, and afterwards terminates in a hard, dark coloured scab.—The form and structure of this Vesicle is peculiar. It's base is circular, or somewhat oval, with a diameter of about four lines on the tenth day. Till the end of the eighth day, its upper surface is uneven, being considerably more elevated at the margin than about the centre, and sometimes indented by one or two concentric furrows, but on the ninth or tenth day the surface becomes plane, and in a very few instances the central part is highest. The margin is turgid, firm, shining, and rounded, so as often to extend a little beyond the line of the base. The Vesicle consists internally of numerous little cells, filled with clear lymph, and communicating with each other. The areola, which is formed round the Vesicle, is of an intense red colour. Its diameter differs in different persons from a quarter of an inch to two inches, and it is usually attended with a considerable tumor and hardness of the adjoining cellular membrane. On the eleventh and twelfth day, as the areola declines, the surface of the Vesicle becomes brown in the centre, and less clear at the margin. The cuticle then begins to separate, and the fluid in the cells gradually concretes into a hard rounded scab of a reddish brown colour. This scab becomes at length black, contracted, and dry, but it is not detached till after the twentieth day from the inoculation. It leaves a permanent circular cicatrix, about five lines in diameter, and a little depressed, the surface being marked with very minute pits or indentations, denoting the number of cells of which the Vesicle had been composed.

" During the progress of the Vesicle some disorder takes place in the constitution, and there is frequently on the arms and back a papulous eruption resembling some forms of the Lichen and Strophulous. These circumstances we should by analogy judge desirable; but they do not always occur, nor are they deemed requisite to ensure the full effect of Vaccine Inoculation, that effect, which, as ascertained and announced by Dr. Jenner, is allowed to be more important than any event which the history of medicine can furnish. He says, " Those persons on whom the Vaccine Vesicle has been excited by perfect matter, and has completely gone through the progressive stages of inflammation, maturation, and scabbing, are ever after secure from the infection of the Small-pox, neither exposure to the Variolous effluvia, nor the insertion of the matter into the skin, producing that distemper." We cannot now withhold our assent to this position generally, since the truth of it has been confirmed by the active experience of the most eminent physicians and surgeons, and by the opinion of other scientific men accustomed to investigation."

Next follow a catalogue of names that have sanctioned the new practice, of which it is enough to say, that they occupy a quarto page and a half, and are all respectable.

The author next remarks the effects of variolous inoculation after vaccination, from the practice of the Small-pox Hospital, and what he he has seen himself. In all these cases the safety of vaccination has been confirmed, and also by experiments at the Foundling Hospital, and at the Vaccine Pock Institution. Some judicious remarks follow, in answer to Mr. Goldson, whose pamphlets we have already considered.

"But (continues Dr. Willan) it is necessary to the advancement of Vaccine Inoculation, or to the reputation of Dr. Jenner, that we should acknowledge his position to be true universally, and invariably? Experienced practitioners will be disposed to answer this question in the negative, since no absolute certainty can be obtained of the precise effects of any medical or chirurgical process. In the infinite diversity of human constitutions, there may be some which are neither susceptible of the Vaccine Disease, nor the Small-pox, others which are susceptible of the former and not of the latter, or vice versa, and others which are susceptible of both at the same time, or, to a certain degree, at separate times: there may also be a few in which the inoculation excites a new mode of action, terminating in Erysipelas, phagedenic ulcer, or other morbid appearances not necessarily connected with the specific disease. Several of these anomalies or exceptions to the general rule have occurred, but certainly not so often as was expected by those who considered the subject, from the first, dispassionately, nor have they been in sufficient number to form any serious objection to the practice founded on Dr. Jenner's discovery. Similar irregularities observed in the constitutions of persons inoculated for the Small-pox, and given to the public in very aggravated statements, did not deter our predecessors from Variolous Inoculation, though, at its commencement, one in forty or fifty died of the communicated disease, or of chronic distempers afterwards.—The too zealous and enthusiastic advocates for the new inoculation, who extended their views far beyond the limits of analogy or probability, have done no service to the cause. Dr. Jenner has expressed his own sentiments with moderation. He thinks "the animal œconomy is precisely under the same laws with respect to the action of Variolous and Vaccine Virus;" hence he concludes that both of them are, by inoculation, preventives of the Small-pox, but that the advantages are greatly in favour of Vaccine Inoculation, because it is equally safe at all ages and in every season, and does not occasion confinement,—because it neither diffuses contagion, nor excites Scrophula, and because it is free from the danger attending the inoculated Small-pox, which still proves fatal in one case out of two hundred and fifty.

"With these concurring circumstances on the side of Vaccine Inoculation, the balance would still remain in its favour, even though its preventive power might be found somewhat less certain than that of Variolous Inoculation. Dr. Jenner, "for argument's sake, not from conviction," puts the question, whether, "if one person in an hundred, after having had the Cow-pox, should be found found susceptible of the Small-pox, this would invalidate the utility of the practice?"—It does not appear that failures in the preventive effect of Vaccine Inoculation, including mistakes, negligences, and mis-statements, have occurred in a greater proportion than as one to eight hundred. Let me then re-state the question, "If one person in a thousand should, after vaccination, be found susceptible of the Small-pox, would the utility of Vaccine Inoculation be invalidated?" Surely not.—I trust, however, that, when the practice has been continued five or six years longer, the number of failing, or anomalous cases, will not exceed the proportion of one in three thousand, and will nearly coincide with the number of failures or anomalies in the inoculated Small-pox. But taking either of the proportions last mentioned, we must acknowledge the advantages of Vaccine Inoculation, and confirm the deduction made from Dr. Jenner's discovery, that the Cow-pox, under proper regulations, affords the means of finally eradicating the Small-pox."

After this follow those remarks on the similarity of the objections against vaccination, to those stated against the first introduction of variolous inoculation. These have been already noticed in our remarks on Mr. Merriman and Dr. Adams.

The third chapter is on Imperfect Vaccination. Of this the author makes three divisions: 1st. When the fluid employed has lost some of its original properties. 2d. When the persons inoculated are soon after affected with any contagious fever. 3d. When they are affected at the time of inoculation with some chronic cutaneous disorders.

Though these subjects have been often discussed, and their importance has induced us to allow them a very large share of our Journal, yet we cannot help complimenting the author on the judicious manner in which he has arranged the most important and best attested facts in illustration of his subject. Several instances are also brought to show, that the same interruption happens to variolous inoculation under similar circumstances. The advantage Dr. Willan has derived from his accurate attention to cutaneous complaints, has enabled him to name as well as describe all the various contagious chronic eruptions which have been found particularly to interrupt this process.

The characters of imperfect vaccination are next attended to; these are divided into pustules, ulcerations, and vesicles of an irregular form. Though it would have been inconsistent with the object of Dr. Willan's performance, to have omitted either, yet as the first and second are not likely to be mistaken by any of our readers who have attended practically to the subject, we shall confine ourselves to the third, remarking only, in general, that the formation of pus or the process of ulceration are inequivocal proofs of failure in the operation.

"I have observed (says Dr. Willan) three sorts of these irregular *Vesicles*. The first is a single pearl-coloured vesicle, set on a hard, dark-red base, slightly elevated. It is larger and more glo-

 bate bate than the pustule above represented, but much less than the genuine vesicle: its top is flattened, or sometimes a little depressed, but the margin is not rounded or prominent. The second appears to be cellular like the genuine vesicle; but it is somewhat smaller, and more sessile, and has a sharp angulated edge. In the first the areola is usually diffuse, and of a dark rose-colour: in the second it is sometimes of a dilute scarlet-colour, radiated, and very extensive, as from the sting of a wasp; at other times it has the form and colour exhibited. The areola appears round these vesicles on the seventh or eighth day after inoculation, and continues more or less vivid for three days, during which time the scab is completely formed. The scab is smaller and less regular than that which succeeds the genuine vesicle; it also falls off much sooner, and, when separated, leaves a smaller cicatrix, which is sometimes angulated.—The third irregular appearance is a vesicle without an areola."

We do not recollect that the *cellular* irregular vesicle has been remarked by any other person, nor does the Doctor direct us to any authorities. It is by far the most important, because the most difficult to be distinguished, of any of the irregularities. We therefore regret the account of it is so short, since engravings, however faithfully executed, can only be considered as auxiliaries to descriptions of local appearances under their progressive forms. As far however as we can judge by the author's further remarks, the constitution is sometimes secured by these irregular vesicles; and where it is not, the fluid they contain will produce a genuine vaccine vesicle in other subjects. It would be a matter of great importance if we could ascertain, that in all those instances in which a mild small-pox had occurred after vaccination, the vesicle had been such as described by Dr. Willan. The following are the author's remarks.

"The effect of Vaccination, when there are irregular Vesicles (pag. 38-9.) is different in different cases. They appear fully to secure some individuals from the infection of the Small-pox, in others the constitution is but imperfectly * guarded against the Small-pox by these Vesicles, the disease taking place after them, at different intervals, under a particular form.† — I may add further, that when the fluid they contain is used for the purpose of inoculation, it sometimes produces an irregular, and at other times a genuine Vesicle. According to Dr. Jenner, "The Vaccine fluid, even in a pustule (vesicle) going through its course perfectly, if taken in its far advanced stages, is capable of producing varieties, which

* Dr. Jenner allows the converse of this position: "Although the susceptibility of the virus of the Cow-pox is for the most part lost in those who have had the Small-pox, yet in some constitutions it is only partially destroyed, and in others it does not appear to be in the least diminished."

† Persons thus partially or imperfectly guarded, seem only to take the disorder after repeated or long-continued exposure to infection.

which will be permanent, if we continue to vaccinate from it."*

If, as Dr. Willan observes, Mr Ring's statement be correct, the fact is very surprizing. But in a matter of so much importance, such an expression as *the eighth or ninth day*, is much too loose. It is well known that many have been safe in whom the progress has not been slower than the above; however, if the only consequences to be apprehended are, that mild small-pox which in some instances has occurred after vaccination, it is of little importance to the patient. The remainder of this chapter consists of some further comparative remarks between vaccination and variolation, and on the propriety of examining the appearance of the cicatrix in all those arms which were vaccinated during the two first years of the practice.

The fourth chapter is on Variolous Eruptions subsequent to Vaccination. As in this we have the advantages of some cases witnessed by the author himself, and described with his usual accuracy, we offer a few of them to the reader, and also the concluding remarks.

"In July 1800, I saw a case of Variolous eruption, six months after Vaccine inoculation, and another about Midsummer, 1801, ten months after vaccination. In both these cases, there was a considerable degree of fever, but the pustules, which were distinct, small, and hard, began to dry off on the sixth day of the eruption. The subjects of them were infants, who took the Small-pox by infection, and as the cicatrix on the arm, was in both instances very slight, I conluded, at that time, that the Vaccine inoculation had wholly failed.

"A third case occurred in the family of Mr. Minton, Banner-square, St. Luke's, which excited much attention. A boy was vaccinated at the age of three months, (March 1802,) by a respectable practitioner, who did not observe any thing particular in the case. Two years afterwards, 4th of March, 1804, this child was affected with sickness at the stomach, heat of the skin, head-ach, and restlessness. The fever continued through the night, and the following day, March 5. In the evening there was an extensive efflorescence, and his parents observed an eruption of red pimples, chiefly on the neck. On the 6th, the rash had disappeared,

* Med. and Phys. Journal, Aug. 1804; also for May 1803, where, however, the remarks seem to have been misprinted.

Compare Dr. Walker's Statement in the Journal for December 1804.

In Mr. Powell's cas (at Chatham,) the inoculation had produced one of the delusive Vesicles above described (pag. 39) if Mr. J. Ring's statement be correct. "It arose at the usual period, and had nothing particular in its appearance, but that it began to die away on the eighth or ninth day, by which time, in general, the Vaccine Vesicle has not arrived at its height. The child took the Small-pox a month afterward. From this patient Mr. Powell inoculated several children, "all of whom were exposed to the contagion of the natural Small-pox, and also were inoculated with Variolous matter, but without effect." Ring's Treatise, p. 599.

peared, but the pimples were numerous on the face, and other parts of the body. On the 5th day of the fever (8th March) some of the eruption became pustular, and was thought to resemble that of the Small-pox, the pustules being indented, having a red base, and containing a whitish fluid. Only a few of them maturated; and a considerable part of the eruption remained hard and papulous throughout the disease. The face and eye-lids were much swollen from the fifth morning to the seventh night, (10th March). On the eighth day of fever, and sixth of the eruption, (11th March) the swelling had subsided, the inflammation had disappeared, the pustules were brown, hard, and dry, and the patient had no further uneasiness. As the eruption, in this case, terminated so speedily, several medical gentlemen were desirous of ascertaining, by inoculation, whether it would produce the Small-pox or some other disease. Accordingly, a sister of the little boy, aged five months, was inoculated in both arms, with matter taken from him on the seventh day of the disease. Two days afterwards, a physician, from what motive I know not, inoculated her with the Vaccine fluid. Both the Variolous and Vaccine inoculation proved effective on the right arm; the Vaccine Vesicle was distinctly formed on the 6th day, and arrived at its acmé on the 10th. The pustule which arose from the other puncture, exhibited the usual appearances after inoculation with variolous matter. The child was affected with fever on the eighth day, and there was an eruption of about eighty pustules on the eleventh and twelfth days. These pustules were hard and conoidal: on the thirteenth day, there was a whitish fluid at their points, and a little redness at their bases. Before the end of the fifteenth day, the redness or inflammation had disappeared, and the pustules were become brown and dry. On the seventeenth and eighteenth day after inoculation, or seventh or eighth of the eruption, all the scabs had separated, leaving the usual marks in the skin.

"The gentleman who vaccinated Master Minton in 1802, did not recollect, after the lapse of two years, the source from which he obtained the fluid. A distinct cicatrix, about 3-10ths of an inch in diameter, remained on the child's arm. He took the disease by infection, at a boarding-school, to which a child had been brought during convalescence from the Small-pox.

"Elizabeth Clark, a distiller's child in Coppice-row, vaccinated May 1800, with fluid taken on the ninth day, was for some time exposed to the contagion of Small-pox, in September 1804. She had considerable fever for two or three days, and then an eruption of small hard pustules, which became brown and dry so soon, that several medical gentlemen, who saw them, could not believe the eruption was variolous. The child's sister, an infant, was therefore inoculated from her, and had an eruption of distinct pustules with fever. In order to prove that this fever and eruption were variolous, the infant was, two months afterwards, inoculated with variolous matter, and exposed to contagion, but without effect."

The

The Fullwood's Rents cases are next detailed, with some others which are communicated. What we have transcribed are sufficient to illustrate the author's meaning, and to prepare the reader for his general inductions, which are introduced by a controversy that does credit to the parties, and to Dr. Willan for producing and imitating it.

"Mr. Dunning observes, respecting these cases,—'That Small-pox of the same benign and modified character will sometimes, but indeed very rarely, happen in persons of highly susceptible habits, placed under exposure to a concentrated and epidemical variolous atmosphere, although they had been previously and duly vaccinated, I am inclined from some late observations to believe, but, thank God, not to dread." On this principle, he invites Mr. Goldson to 'join the vaccine party with his strong forces, and assist in diffusing a national blessing.' We shall perhaps find, through the experience of other practitioners, that the observations of Mr. Dunning and Mr. Goldson virtually coincide, and we need not yet despair of an union in the cause of Vaccination, between these eminent surgeons, whose abilities and active services are so highly esteemed in our two great sea-ports."

Dr. Willan next adverts to the argument that vaccination may be a security for a time only, which he very properly dismisses as unsupported by fact or analogy. Mr. Goldson's suggestion that the occurrence of some other contagious eruption after vaccination, may restore the constitutional susceptibility to the small-pox, is shown by several instances to be equally fallacious.

The subject of small-pox exposure after vaccination being thus amply discussed, some remarks follow on the various effects of of small pox *inoculation* in persons who have been previously vaccinated. By these it appears, that the effects have been exactly similar from inoculation for small-pox, in such as have gone through the disease in a casual way, or by inoculation. Some general remarks follow, shewing that nothing which has hitherto occurred should lessen our confidence in this invaluable discovery; and the chapter concludes with an account of Mr. Brices's proposal for a second vaccination on the 4th or 5th day after the first, in which the second puncture ought to be accelerated, so to begin drying at the same time as the first. This was originally introduced by Dr. Woodville, in the small-pox inoculation. Dr. Willan very properly remarks, that, under such circumstances, the lymph for the second vaccination should not be taken from the patient's arm when the primary vesicle is one of the irregular kind, above described, which produces disorder of the constitution, but only an imperfect security against the small-pox.

The fifth chapter is on the "cutaneous and glandular affections imputed to vaccine inoculation." In this we have the result of his own occular inspection to satisfy us, that what cutaneous eruptions have hitherto been imputed to cow-pox, were known from the period of the earliest writers; and that the number of cutaneous cutaneous diseases, or of cases, has not increased since the introduction of cow-pox. Mr. Trye's valuable communication is inserted from our Journal, shewing that the people employed in the Gloucestershire dairies are among the healthiest in the country. The chapter concludes with a general remark, that glandular affections very rarely follow vaccination; and always, in a slight degree; that they are comparatively more frequent after variolation, and much more serious.

A very important chapter follows on a disease, which, on account of its comparative mildness, has been too much neglected by medical writers. The late controversies have brought chicken-pox into much greater notice than it merited, when Dr. Hebberden thought it worthy of a place in the Medical Commentaries; and our readers will readily believe Dr. Willan has exerted his usual diligence and accuracy on the occasion. On this account, we shall forbear to make any extract, as those who wish for information will consult the whole.

Such is the abstract of this work, which the reader will at once see, contains all that has been written of any importance on this subject, arranged and digested by one so competent to the undertaking, and every where enriched by remarks from the same source. If less original matter is contained in it than in Dr. W's other performances, it must be imputed to the numerous communications which our Journal has enabled every medical and scientific writer to offer with so much ease, and with a certainty of general circulation. Though, by these means much has been known, yet such a selection as the present was much wanted, and we know of no one so fit to make it as the author of the Treatise on Cutaneous Eruptions.

